# Head Lice: An Under-Recognized Tropical Problem

**DOI:** 10.4269/ajtmh.17-0656

**Published:** 2017-12-06

**Authors:** Suny Coscione, Christian Kositz, Michael Marks

**Affiliations:** 1 Clinical Research Department, Faculty of Infectious and Tropical Diseases, London School of Hygiene & Tropical Medicine, London, United Kingdom;; 2 The Hospital for Tropical Diseases, Mortimer Market Centre, Mortimer Market, London, United Kingdom

**Figure 1.**
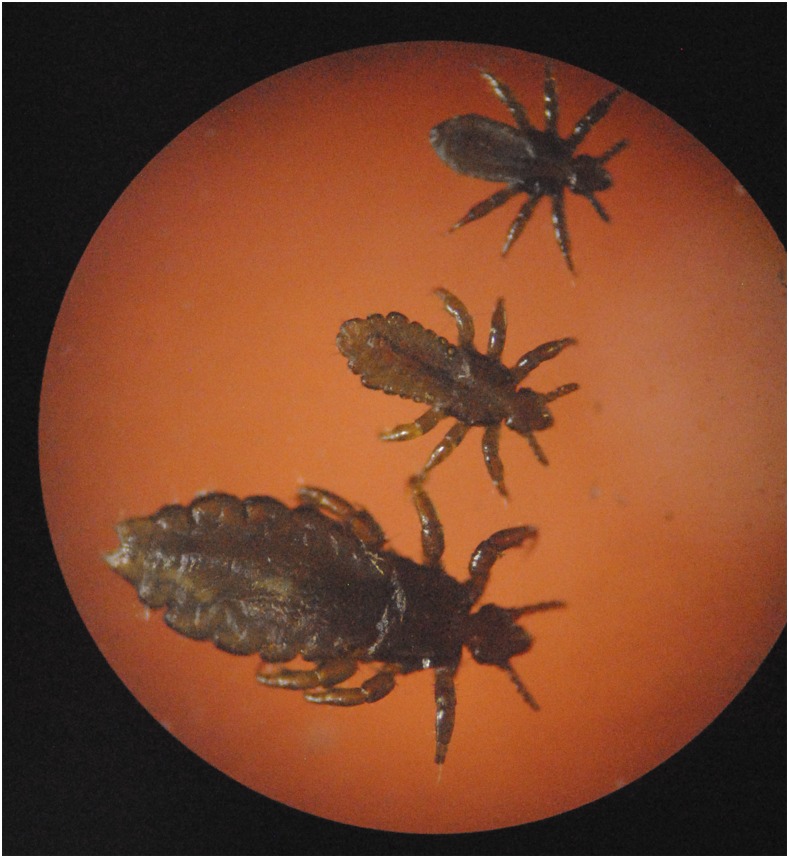
Nymph and adult stages of *Pediculus humanus capitis*. This figure appears in color at www.ajtmh.org.

**Figure 2.**
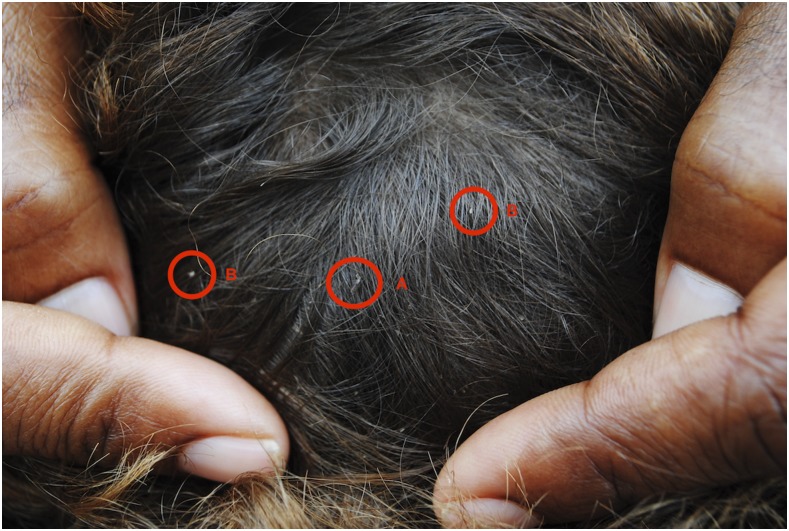
Examination of an individual’s hair containing both an adult louse (A) and eggs (B). This figure appears in color at www.ajtmh.org.

**Figure 3.**
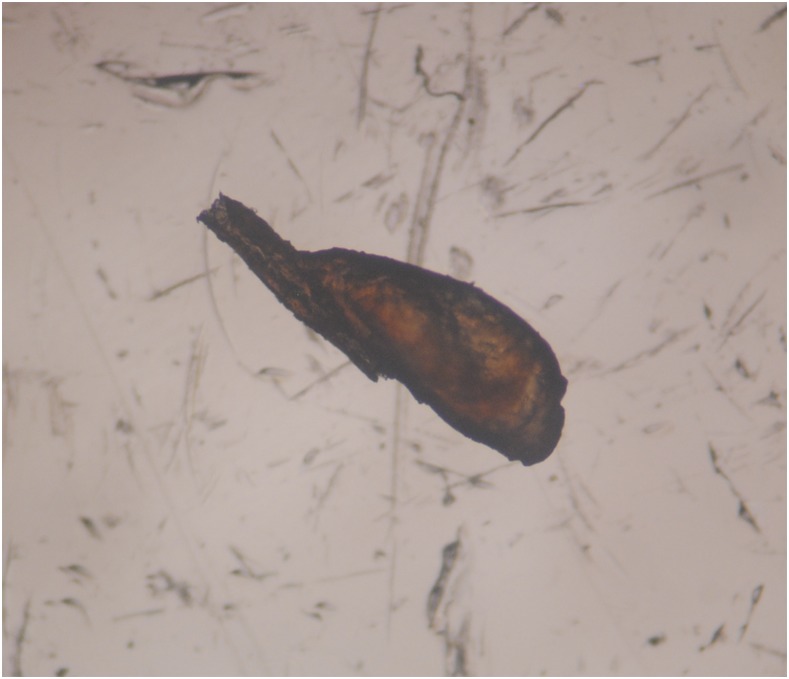
Microscopic image of an egg (nit) attached to a human hair. This figure appears in color at www.ajtmh.org.

Head lice, caused by infestation with *Pediculus humanus capitis*, is an extremely common problem in tropical countries. *Pediculus humanus capitis* is an obligate human ectoparasite. Morphologically, head lice are indistinguishable from *Pediculus humanus corporis*, the human body louse, although they are slightly smaller. Unlike body lice, head lice have not clearly been proven to be vectors for infectious agents. Adult head lice develop through three nymphal stages (Figure 1) and feed on blood from the scalp two to six times a day causing discomfort and pruritus. On examination, the eggs (nits) are more commonly identified than adult lice (Figure 2). The complete life cycle takes 15–20 days, and adults survive up to 1 month. Adults mate once, and a fertilized female then produces 3 to 4 eggs per day (Figure 3) for the remainder of their lives. Nymphs must feed immediately on hatching, and therefore, nits located more than 1 cm from the scalp are considered nonviable. Infestation results in distress, social stigma, and absence from school.^1^ Like other ectoparasitic infections, the prevalence of head lice may be high amongst children in remote and rural settings.^2^ In these settings, access to treatment is frequently limited, and many individuals rely on traditional medicine. There is increasing resistance to pyrethroids and malathion, the most commonly used first-line topical agents.^3^ More recently, both oral and topical ivermectin^3,4^ have shown promise for treating head lice, but access to these drugs to treat head lice is nonexistent in low-income settings. Mass treatment of scabies, onchocerciasis, or lymphatic filariasis might have an impact on head lice although data specifically examining this hypothesis are lacking, and there is a risk that resistance to ivermectin might develop.^5^
